# Antihypertensives associated adverse events: a review of mechanisms and pharmacogenomic biomarkers available evidence in multi-ethnic populations

**DOI:** 10.3389/fphar.2023.1286494

**Published:** 2023-12-01

**Authors:** Sahar M. Altoum, Zeina N. Al-Mahayri, Bassam R. Ali

**Affiliations:** ^1^ Department of Genetics and Genomics, College of Medicine and Health Sciences, United Arab Emirates University, Al Ain, United Arab Emirates; ^2^ ASPIRE Precision Medicine Research Institute Abu Dhabi, United Arab Emirates University, Al Ain, United Arab Emirates

**Keywords:** antihypertensives, hypertension, adverse events, side effects, pharmacogenomics, toxicity, mechanisms of adverse events

## Abstract

Hypertension remains a significant health burden worldwide, re-emphasizing the outstanding need for more effective and safer antihypertensive therapeutic approaches. Genetic variation contributes significantly to interindividual variability in treatment response and adverse events, suggesting pharmacogenomics as a major approach to optimize such therapy. This review examines the molecular mechanisms underlying antihypertensives-associated adverse events and surveys existing research on pharmacogenomic biomarkers associated with these events. The current literature revealed limited conclusive evidence supporting the use of genetic variants as reliable indicators of antihypertensive adverse events. However, several noteworthy associations have emerged, such as 1) the role of *ACE* variants in increasing the risk of multiple adverse events, 2) the bradykinin pathway’s involvement in cough induced by ACE inhibitors, and 3) the impact of *CYP2D6* variants on metoprolol-induced bradycardia. Nonetheless, challenges persist in identifying biomarkers for adverse events across different antihypertensive classes, sometimes due to the rarity of certain events, such as ACE inhibitors-induced angioedema. We also highlight the main limitations of previous studies that warrant attention, including using a targeted gene approach with a limited number of tested variants, small sample sizes, and design issues such as overlooking doses or the time between starting treatment and the onset of adverse events. Addressing these challenges requires collaborative efforts and the integration of technological advancements, such as next-generation sequencing, which can significantly enhance research outcomes and provide the needed evidence. Furthermore, the potential combination of genomic biomarker identification and machine learning is a promising approach for tailoring antihypertensive therapy to individual patients, thereby mitigating the risk of developing adverse events. In conclusion, a deeper understanding of the mechanisms and the pharmacogenomics of adverse events in antihypertensive therapy will likely pave the way for more personalized treatment strategies to improve patient outcomes.

## 1 Introduction

Hypertension (HTN) significantly contributes to cardiovascular disease, impacting roughly 42% of adults globally (Hypertension, 2023). The first-line therapies used to reduce blood pressure (BP) include angiotensin-converting enzyme inhibitors (ACEIs), angiotensin receptor blockers (ARBs), thiazide and thiazide-like diuretics, calcium channel blockers (CCBs), and beta (β) blockers (BBs) (McDonough et al., 2021). The choice among available antihypertensives (AHs) hinges on specific indications, restrictions, comorbidities, and various patient-related factors (JSH, 2014). Per the guidelines of the American College of Cardiology and American Heart Association (ACC/AHA), an agent from the aforementioned groups is recommended when a patient qualifies for drug therapy except when a comorbidity is present and requires a specific AH class. For example, CCBs are favored in post-renal transplant patients (Flack and Adekola, 2020). In addition, ethnicity is a crucial consideration when prescribing AHs. For instance, Black Americans are more susceptible to resistant and nighttime HTN, stroke, and heart failure (HF), and they experience HTN and associated organ damage at younger ages. Consequently, the treatment for these patients usually involves a combination tablet of thiazide-like diuretic or ARB with CCBs as the first-line treatment (Unger et al., 2020).

The pharmacological treatment of HTN has shown effectiveness in reducing HTN-related morbidities. However, a persistent issue in managing HTN is medication non-adherence. Contributing to this non-adherence is the chronic and often asymptomatic nature of HTN, where lack of immediate apparent symptoms leads some patients to sporadically or regularly skip the prescribed medications (Hamrahian et al., 2022). Medication adherence varies throughout therapy and tends to decline over time. Globally, the incidence of non-adherence to AH medications among HTN patients ranges from 27% to 40% (Lee et al., 2022). The World Health Organization (WHO) has identified five categories of obstacles affecting drug adherence, including patient demographics, issues within the healthcare system, concerns related to therapy such as medication adverse effects, disease-related factors, and patient-related challenges, including the fear of adverse effects (Hamrahian et al., 2022). For instance, the highest cause of non-adherence to diuretics may be explained by their side effects, which directly affect the quality of life (Gupta et al., 2017).

As a potential solution to enhance AH adherence, personalized treatment within a patient-centered approach is suggested (Hamrahian et al., 2022). This personalization can be achieved by considering interindividual environmental, lifestyle, and genetic differences and their effect on pharmacological response. As such, pharmaco-metabolomics, pharmaco-mirobiomics, and pharmacogenomics have evolved to explain factors leading to heterogeneous drug responses and to optimize pharmacological therapies (Zhao et al., 2023). Pharmacogenomics, which involves genomic biomarkers for customizing drug choices and dosages, offers an alternative to the conventional “one size fits all” approach (Primorac et al., 2020). Numerous research groups have explored pharmacogenomics biomarkers for AH drug therapy outcomes (Xiao et al., 2022), primarily concentrating on drug response outcomes (Hiltunen et al., 2015; Eadon et al., 2018; Oliveira-Paula et al., 2019). The intricate nature of HTN control contributes to the challenge of identifying clinically applicable response biomarkers for the AH personalization (Eadon et al., 2018). Although a few studies proposed that genetic variation may account for a significant proportion of adverse events associated with AH therapy, there has yet to be a comprehensive review evaluating the available evidence from these research endeavors.

The current review evaluates research exploring pharmacogenomics biomarkers and possible AH therapy-associated adverse events mechanisms. In the subsequent sections, we delineate the adverse effects of AH agents, their suggested molecular mechanisms, and the research findings concerning pharmacogenomic biomarkers linked to these events.

## 2 Angiotensin-converting enzyme inhibitors

**FIGURE 1:**
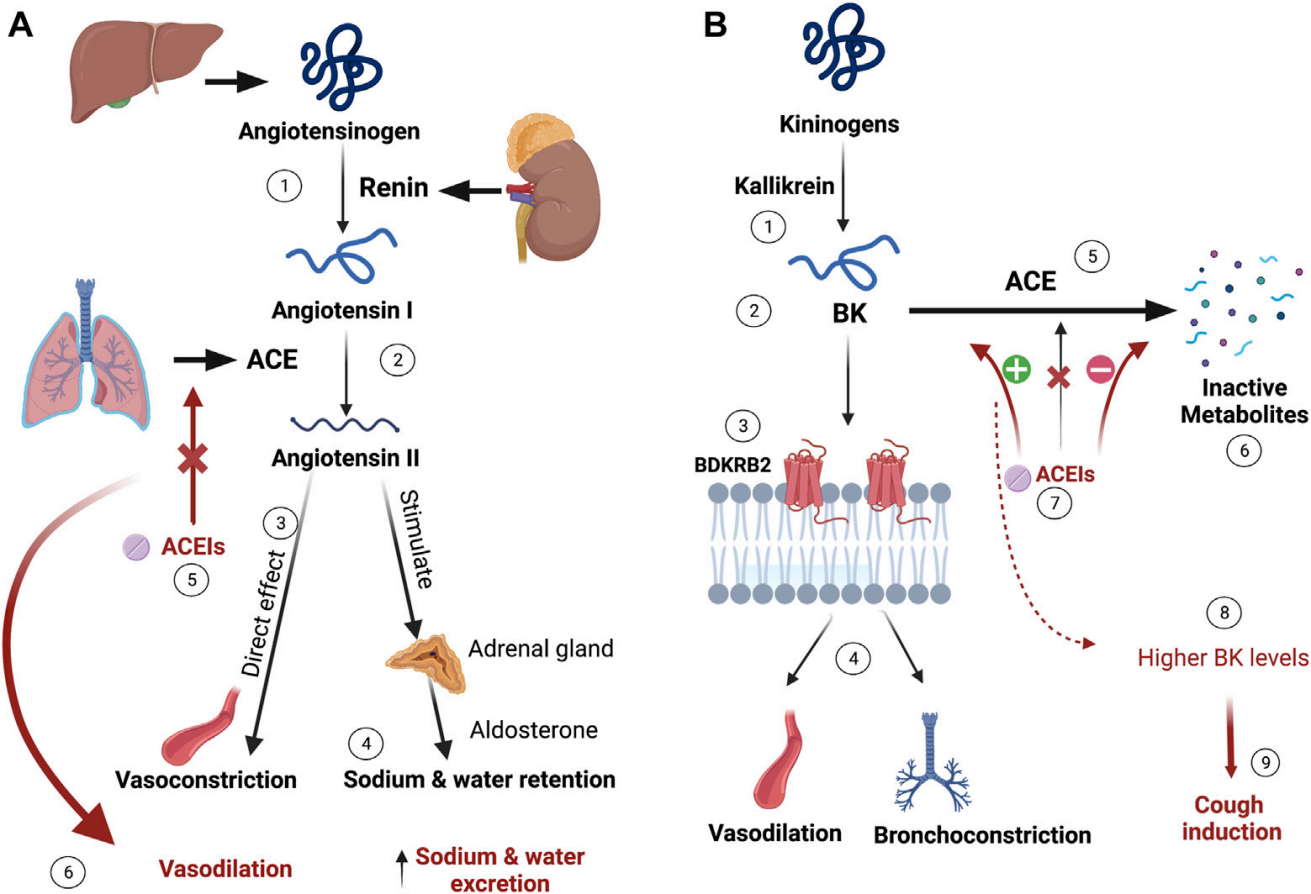
ACEIs mechanism of action and proposed ACEIs-induced cough mechanism. **(A)** Liver synthesizes angiotensinogen, which is converted to angiotensin-I by kidney-produced renin. The conversion of the inactive angiotensin-I into the vasoconstrictor angiotensin-II is catalyzed by the angiotensin-converting enzyme (ACE) produced mainly from the lungs. Besides the direct vasoconstrictive effects of angiotensin-II, it exerts sodium and water retention by stimulating aldosterone release from the renal gland. ACE inhibitors (ACEIs) prevent this process resulting in vasodilation and sodium and water excretion. **(B)** Bradykinin (BK), produced by the kallikrein activation of kininogens, binds to Bradykinin 2 receptor (BDKRB2), exerting vasodilation and bronchoconstriction effects. BK is metabolized to its inactive metabolites by ACE. When ACEIs inhibit ACE, higher levels of BK accumulate and bind to BDKRB2, which is thought to be causing irritation and cough. (Figure created with BioRender.com).

ACEIs are frequently prescribed for blood pressure management and reducing cardiovascular events such as myocardial infarction and HF. Agents such as lisinopril, enalapril, and perindopril function by regulating the renin-angiotensin-aldosterone System (RAAS), significantly impacting the cardiovascular system. ACEIs work by inhibiting angiotensin-converting enzyme I, thereby preventing the conversion of angiotensin I into the potent vasoconstrictor angiotensin II (Figure 1A). This group is usually well tolerated among patients from different populations. However, one of its most common side effects is ACEIs-induced dry cough (ACEIs-cough), which limits their use and often results in drug discontinuation. This dry cough affects 5%–35% of treated patients, primarily females (Mukae et al., 2000; Moholisa et al., 2013; Luo et al., 2015; Mosley et al., 2016; Hallberg et al., 2017). Cough may develop within days to several months after starting the treatment and typically resolves within 1 week to 3 months after discontinuation. Additionally, ACEIs may prompt ACEIs-induced angioedema (ACEIs-AE), an infrequent yet life-threatening side effect characterized by swelling in the face, lips, tongue, and skin layers. In severe cases, it can lead to suffocation and even fatalities (Moholisa et al., 2013).

To date, the exact mechanism behind ACEIs-cough remains unclear. The prevailing suggestion is that specific mediators, like bradykinin (BK) and substance P, potentially contribute to inducing cough. BK is primarily broken down by ACE and, to a lesser extent, by the aminopeptidase P (APP) enzyme. When ACEIs inhibit the ACE enzyme, it leads to an increase in BK availability, triggering pathological responses such as bronchoconstriction, swelling, and cough (Moholisa et al., 2013; Luo et al., 2015; Mosley et al., 2016; Hallberg et al., 2017). The proposed mechanism for ACEIs-induced cough is illustrated in Figure 1B. Numerous studies have explored the potential for a genetic predisposition to developing this side effect.

One of the earliest studies on this topic investigated the association of variants in the bradykinin receptor B2 (*BDKRB2*) gene with ACEIs-cough. The findings indicated a strong association between rs1799722 (g.96204802C>T) in the *BDKRB2* promoter region and ACEIs-cough. Patients with TT genotype at this variant were more susceptible to experiencing cough compared to CC genotype carriers, particularly noticeable in females. Previous experiments demonstrated that the same variant leads to higher transcriptional activity of BDKRB2. Therefore, the authors suggested that individuals with this variant have an increased availability of receptors for BK to exert its effects, allowing for heightened BK activity, consequently resulting in cough (Mukae et al., 2000). The association between *BDKRB2:*rs1799722 and ACEIs-cough was not replicated in another study. However, the latter study was conducted in a group of South African patients, contrasting the earlier study involving Asians. Nonetheless, the same study reported a different association between cough incidence and a nine-base-pair deletion in the *BDKRB2* first exon, referred to as the B2 -9 allele. The results highlighted a significant association between B2-9 and ACEIs-cough (Moholisa et al., 2013).

On the other hand, multiple groups examined the effect of *ACE* gene variation on ACEIs-cough. A common variant, rs1799752 (g.63488543_63488544ins-11T-GAGACGGAGTCTCGCTCTGTCGCCCATACAGTCACTTTT), has shown significant results. This variant is an insertion (Ins)/deletion (Del) characterized by the presence or absence of 287 bp “Alu repeat,” which is known to affect the ACE serum activity. Patients carrying the homozygous deletion (Del/Del) have higher ACE serum activity in contrast to those with heterozygous (Ins/Del) and homozygous insertion (Ins/Ins) carriers, with intermediate and low ACE activity, respectively. The two latter groups are found to accumulate BK, which ACE degrades. Accordingly, patients in these groups are at more risk for ACEIs-cough than Del/Del carriers. This association was more significant in Asians compared to Caucasians (Ding et al., 2000; Niu et al., 2002; Li et al., 2012). Mu et al. (2019) applied a meta-analysis of studies that examined genetic variants in *ACE* and/or *BDRKB2* in association with ACEIs-cough. The analysis included 26 studies, with 1,641 cough cases versus 1,559 controls. The results show a significant association between *ACE*: Ins/Ins genotype and the induced cough. The different distribution of this variant among ethnicities might explain the inconsistent results found in the analyzed studies. However, the meta-analysis proved that ACE Ins/Del could be a promising biomarker for predicting ACEIs-cough, specifically for East Asians (Mu et al., 2019). In contrast, one study found that rs4459610 (g.63507359A>T) and rs4267385 (g.63506395C>T) located on *ACE* are protective against ACEIs-cough. Patients with at least one of the reference alleles had a lower chance of developing cough (Mas et al., 2011).

Moreover, one study focused on solute carrier organic anion transporter 1B1 (*SLCO1B1*) variants and their possible contribution to enalapril-induced cough. Enalapril is one of the ACEI cleared through the hepatic transporter encoded by *SLCO1B1*. Luo et al. (2015) investigated two common *SLCO1B1* variants. They found that among Chinese patients taking enalapril *SLCO1B1*: rs4149056 (g.21178615T>C) −521C alleles resulted in a 2-fold relative risk of induced cough, which increases to 6.94-fold in SLCO1B1*15/*15 carriers. The authors proposed that reduced hepatic drug uptake in patients with defective *SLCO1B1* alleles resulted in increased blood concentration of active enalaprilat and heightened the risk of cough (Luo et al., 2015).

Finally, studies correlated the presence of *ABO* gene variants with ACEIs-cough. The *ABO* gene encodes the glycosyltransferase enzyme, which significantly influences ACE glycosylation, affecting ACE’s glycoprotein stability, folding, intracellular targeting, and resistance to proteolysis (Ding et al., 2000; Niu et al., 2002). Patients with one or two *ABO:*rs495828 (g.133279294T>G) reference alleles exhibit reduced ACE activity due to ACE glycosylation disruption (Anthony et al., 2010; Gassó et al., 2014; Chaikan and Malisorn, 2020), which may explain the observed association of this variant with ACEIs-cough (Mas et al., 2011). Another study involving Chinese patients taking enalapril concluded that the association between *ABO*:rs495828 and ACEIs-induced cough was predominantly noticeable in females (Luo et al., 2014).

**FIGURE 2:**
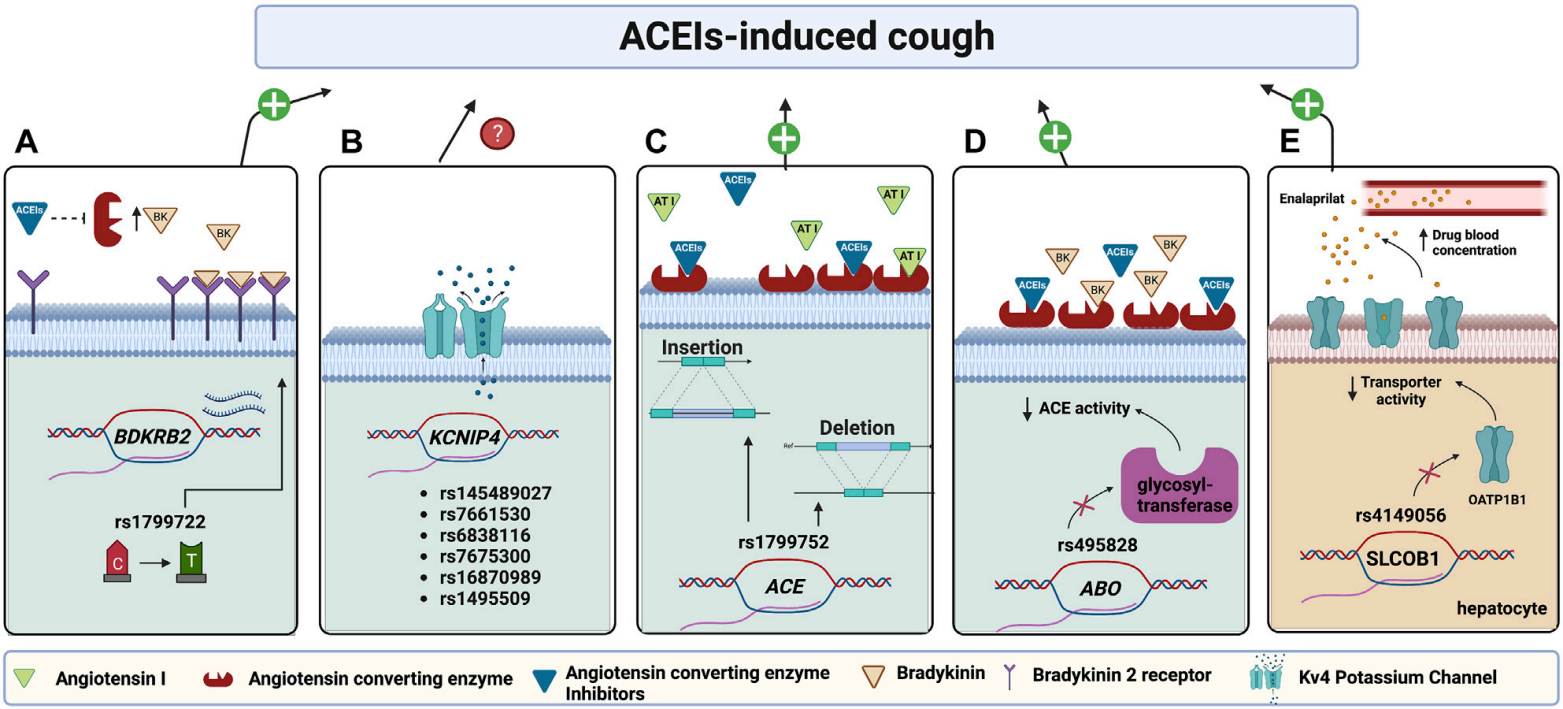
Pharmacogenes found to be associated with ACEI-induced cough and their proposed mechanisms. **(A)** A variant in the Bradykinin 2 receptors (*BDKRB2*) promotor increases its transcription, thus increasing the receptors’ abundance; hence when ACE is inhibited by ACEIs, an elevated bradykinin binding causes bronchoconstriction and cough. **(B)** Variants in potassium channel-interacting protein 4 (*KCNIP4*) encoding for KCNIP4 are associated with ACEI-induced cough with an unknown mechanism. **(C)** Variants in *ACE* affect the enzyme levels. The low enzyme level in the ACE insertion allele (Ins), which will be further suppressed by ACEI, will not be enough to metabolize bradykinin leading to inducing cough. The opposite case applies to deletion allele (Del) carriers. **(D)** A variant in *ABO* encoding glycosyltransferase enzyme affects ACE glycosylation resulting in decreasing ACE activity which will be further suppressed by ACEIs, thus, will not be enough to metabolize BK resulting in its accumulation and inducing cough. **(E)** A variant in *SLCO1B1,* encoding hepatocyte OATP1B1 transporter, decreases transporter activity resulting in decreasing enalaprilat transportation into hepatocytes for metabolism. Accordingly, enalaprilat accumulates in the blood resulting in a more cough risk. (Figure created with BioRender.com).

Other groups applied genome-wide association studies (GWAS) to investigate genetic variants associated with ACEIs-cough. Mosley et al. (2016), for example, performed a GWAS for 1,595 cases of ACEIs-cough versus 5,485 controls (no cough). The results illustrated significant associations between ACEIs-cough development and six variants located on intron 4 of the Kv Channel Interacting Protein 4 (*KCNIP4*) gene. Among these six SNPs, allele “A” at rs145489027 (g.21389571G>A) showed the strongest association. The mentioned SNP, along with other SNPs, namely, rs7661530 (g.21349637T>C), rs6838116 (g.21354260A>C), rs7675300 (g.21383914C>A), rs16870989 (g.21385141T>A), and rs1495509 (g.21391993T>C), exhibited a significant association, with odds ratio ranging from 1.17 to 1.27, in patients mostly of European ancestry. This study has strengths related to its large sample size and design, which involved replication in two independent cohorts and a meta-analysis (Mosley et al., 2016). Ironically, another GWAS on Swedish patients (124 ACEIs-cough cases and 1,345 no cough controls) showed no significant association between *KCNIP4* variants and ACEIs-cough; nevertheless, the findings supported a role of neural activity in ACEIs-cough. The same study identified other candidate variants; however, none reached the genome-wide significance level. The authors speculated that the few cases and not controlling for cough onset are behind the lack of significant associations in their cohort (Hallberg et al., 2017). The exact mechanism by which the variants in *KCNIP4* induce ACEIs-cough is still controversial, as this channel is not expressed in the lungs but is prominently expressed in the brain. It is hypothesized that due to ACE inhibition by ACEIs, the inflammatory mediators usually degraded by ACE are accumulated in the lungs; thus, they stimulate the sensory afferent neurons, resulting in lung hyperresponsiveness and cough (Mosley et al., 2016). Figure 2 illustrates the pharmacogenes associated with ACEIs-cough and their proposed mechanisms.

As mentioned earlier, angioedema is a rare and severe side effect of ACEIs. A few studies have aimed to pinpoint the genetic factors behind ACEIs-AE. These studies identified a significant association between ACEIs-AE and a variant in the X-Prolyl Aminopeptidase 2 (*XPNPEP2*) gene. *XPNPEP2* encodes APP enzyme, which is minorly involved in the degradation of BK and its active metabolite. When ACEIs inhibit ACE activity, APP assumes a more significant role in their degradation. rs3788853 (g.129736814C>A) polymorphism, associated significantly with ACEIs-AE, decreases APP enzyme activity. Combined with ACE inhibition, this reduction leads to the accumulation of mediators, resulting in ACEIs-AE (Duan et al., 2005; Woodard-Grice et al., 2010). Given that *XPNPEP2* is located on the X-chromosome, this association is more evident in men than in women who might possess a normal copy of the gene (Woodard-Grice et al., 2010; Flaten and Monte, 2017).

Another group examined the exons and regulatory regions of *XPNPEP2*. Three polymorphisms, rs3788853 (g.129736814C>A), rs2050011 (g.129737601G>T), and (393G>A), in the regulatory region of the gene, were found to reduce the enzyme activity and levels by influencing the gene’s transcription regulation. A haplotype analysis revealed that patients with a haplotype ATG at the three SNPs, respectively, had significantly lower plasma APP enzyme activity, indicating a functional effect of this haplotype on APP promoter transactivation. This haplotype was significantly associated with ACEIs-AE (Cilia La Corte et al., 2011). However, contrasting outcomes emerged in other studies where certain patients receiving ACEIs, despite having low APP activity, did not develop ACEIs-AE. This disparity suggests the potential involvement of additional genetic and environmental factors (Duan et al., 2005). Interestingly, the same genes investigated for ACEIs-cough were also scrutinized for ACEIs-AE without significant associations being detected (Gulec et al., 2008; Bas et al., 2010; Moholisa et al., 2013).

On the other hand, one study examined the pharmacogenomic associations of ACEIs-AE in a GWAS. Although no associations with genome-wide significance were detected, two associations with modest significance were reported. rs500766 (g.6508628C>T) in protein kinase C theta (*PRKCQ*) was found to decrease the risk of ACEIs-AE if the patient carries at least one copy of the alternative allele. In contrast, the “G” allele at rs2724635 (g.11747039G>A) on ETS variant transcription factor 6 (*ETV6*) was associated with an increased risk for ACEIs-AE. Both genes are involved in immune system regulation, but their role in ACEIs-AE pathogenesis is unclear. A candidate-gene approach analysis in the same study identified rs989692 (g.155083576T>C) on the membrane metalloendopeptidase (*MME*) gene to be associated with ACEIs-AE. *MME* encodes neprilysin enzyme, which is involved in BK and substance P degradation (Pare et al., 2013).

An additional GWAS with the same objective was carried out in Sweden; however, it failed to replicate the same associations (Rasmussen et al., 2020). This GWAS identified variants in intron 1 of the calcium-activated potassium channel subunit alpha-1 (*KCNMA1*) as significantly associated with ACEIs-AE. On a genome-wide level, six variants in this gene showed significant associations: rs2253201 (g.77596639G>A), rs2253202 (g.77596635G>A), rs2673471 (g.77597565A>G), rs2619635 (g.77598844G>A), rs2670121 (g.77599131A>G), and rs2673455 (g.77599353C>G). *KCNMA1* encodes the pore-forming alpha subunit of a potassium channel crucial in cell membrane repolarization. Although this study did not explore the functional activity of the variants, motif analysis of variants in high Linkage Disequilibrium (LD) indicated a potential alteration in the binding site of tissue-specific transcription factors. This alteration might impact the regulation of distant genes, including *KCNMA1* (Rasmussen et al., 2020).

For the first time, one group utilized whole exome sequencing to investigate genetic biomarkers of angioedema. An association of factor V (*F5*) variants, active in blood coagulation, and angioedema induced by ACEIs, or ARBs, were identified. The strongest association was with the common factor V Liden, rs6025 (g.169549811C>T) variant. However, the association signal was more robust when other rare variants in the same gene, namely, rs200157005 (g.169530994G>A), rs149389480 (g.169542338G>A), rs143509841 (g.169555304T>G), and rs140530655 (g.169544331C>T), and chr1:169529903 were added. Notably, individuals with Hereditary Angioedema (HAE) type III have mutations in the coagulation factor XII (*F12*) gene, responsible for encoding factor XII. Factor XII plays a role in releasing BK by activating the prekallikrein enzyme, subsequently initiating a sequence of interactions resulting in BK release. Mutations in the *F12* increase prekallikrein enzyme activity, leading to increased BK production. Consequently, the authors proposed that mutations in other coagulation factors may produce similar effects in causing ACEIs-AE (Maroteau et al., 2020).

Insufficient evidence currently supports the preemptive testing of identified genetic variants as indicators for a high risk of ACEIs-associated adverse events. The strongest association to date for ACEIs-cough is with *ACE* variants. Moreover, the accumulated evidence of the BK pathway involvement in the ACEIs-cough mechanism indicates that more investigation of genes active in this pathway, including *BDKRB2*, is needed. Despite the mild nature of this adverse event, it affects drug adherence and treatment outcomes. On the other hand, ACEIs-AE is a severe and potentially life-threatening adverse event. Therefore, identifying biomarkers of vulnerability is crucial to reduce its occurrence rates. Next-generation sequencing has facilitated the identification of *F5* variants as potential contributors to vulnerability to this adverse event. Future investigations should prioritize collaborative efforts to gather more cases experiencing such rare events to enhance the detection power of associations. Additionally, technical advancements can strengthen these investigations, validating the identified associations and uncovering other unforeseen genetic factors.

## 3 Angiotensin II receptor blockers

ARBs are commonly used as ACEIs alternatives for treating HTN, HF, and chronic kidney disease (CKD). ARBs, including losartan and valsartan, modulate RAAS and inhibit the vasoconstrictor angiotensin II binding to angiotensin II-1 (AT1) receptors in the vascular smooth muscle resulting in vasodilation (Hill and Vaidya, 2022). Given their non-inferior effect, ARBs are increasingly used, even if patients were not tried on ACEIs. Nevertheless, limited evidence exists about the ARBs association with increased myocardial infarction incidence after prolonged use (Lee et al., 2023). Regardless of this unresolved event, this group of medications is well tolerated, and its typical side effects include dizziness, hypotension, and rarely angioedema (Hill and Vaidya, 2022). Kidney failure is another reported adverse event (Hill and Vaidya, 2022); nevertheless, there is evidence that the benefits of using ARBs or ACEIs can reduce mortality rates following an acute kidney injury (Chen et al., 2021).

ARBs-induced angioedema (ARBs-AE) occurs in approximately 0.03% of treated patients, with a higher incidence noted among individuals of African descent and those with a family history (Maroteau et al., 2020). Unlike ACEIs, ARBs are believed to have minimal impact on BK levels. Consequently, the precise mechanism by which ARBs can cause AE remains elusive (Gulec et al., 2008).

Due to its rare occurrence, limited studies have explored the potential genetic predisposition to develop AE. Indeed, the studies investigating ACEIs-AE included some patients who developed AE with ARBs. *KCNMA1* variants [rs2253201 (G>A), rs2253202 (G>A), rs2673471 (A>G), rs2619635 (G>A), rs2670121 (A>G), and rs2673455 (C>G)], and *F5* variants [rs6025 (g.169549811C>T), rs200157005 (g.169530994G>A), rs149389480 (g.169542338G>A), rs143509841 (g.169555304T>G), and rs140530655 (g.169544331C>T)] yielded significant associations with AE in patients using ARBs (Maroteau et al., 2020; Rasmussen et al., 2020). Nevertheless, due to the limited number of patients studied, there is insufficient data to support these associations firmly, and further studies—preferably conducted within collaborative international networks—are required. Table 1 outlines the major studies identifying statistically significant associations between pharmacogenetic variants and adverse events related to ACEIs or ARBs.

**TABLE 1:** Studies investigating pharmacogenomic biomarkers associated with ACEIs and ARBs-induced adverse events.

	rs ID	Variant locus (GRCh38)	Sample	Ethnicity	Medication	Studied outcome	Genotype or allele-association with the—outcome	*p*-value	Reference
*BDKRB2*	rs1799722	g.96204802C>T	260 (30 with ACEI-cough)	East Asian	ACEI	Cough	TT genotype—increased cough risk	.001	Mukae et al. (2000)
	—	9-basepair repeat deletion in exon 1 (referred to as B2-9)	165 (52 ACEI-cough, 36 ACEI-AE)	Mixed	Enalapril	Cough	B_2_-9 (as dominant allele)-increased cough risk	.02	Moholisa et al. (2013)
						Angioedema	B_2_-9 (as dominant allele)-increased angioedema risk	.008	
*ACE*	rs1799752	g.63488543_63488544	Meta-analysis (total 1,641 cases and 2,536 controls)	Mixed	ACEI	Cough	Ins/Ins genotype—increased cough risk (Pooled OR = 1.72, CI 1.52–2.37)	.001	Mu et al. (2019)
		ins-11TGAGACGGAGTCTCGCTCTGTCGCCCATACAGTCACTTTT							
	rs4459610	g.63507359A>T	281, 102 cases and 179 controls)	European	unspecified ACEI	Cough	AA + AT genotypes are associated with a protective effect against cough	.005	Mas et al. (2011)
	rs4267385	g.63506395C>T					CC + CT genotypes—protective effect against cough	.004	
*ABO*	rs495828	g.133279294T>G					Allele T—increased risk of cough	.001	
			450	East Asian (China)	Enalapril	Cough	TT—increased risk of cough	.018	Luo et al. (2014)
*SLCO1B1*	rs4149056	g.21178615T>C	450	East Asian (China)	Enalapril	Cough	Allele C–2-fold relative risk	6.2 × 10^−4^	Luo et al. (2015)
	*SLCO1B1* ^a^15						SLCO1B1^a^15/^a^15–6.94-fold relative risk	.02	
*KCNIP4*	rs145489027	g.21389571G>A	7,080 (1595 ACEI-cough, 5,485 controls)	Mixed but the majority Europeans	Any ACEI	Cough	Allele A—increased risk	1.0 × 10^−8^	Mosley et al. (2016)
	rs7661530	g.21349637T>C					Allele T—increased risk	6.1 × 10^−8^	
	rs6838116	g.21354260A>C/T					Allele A—increased risk	3.1 × 10^−8^	
	rs7675300	g.21383914C>A					Allele A—increased risk	1.1 × 10^−7^	
	rs16870989	g.21385141T>A					Allele A—increased risk	1.1 × 10^−7^	
	rs1495509	g.21391993T>C					Allele C—increased risk	7.8 × 10^−8^	
*XPNPEP2*	rs3788853	g.129736814C>A	566 (169 cases, 397 controls)	African and European Americans (50%:50%)	unspecified ACEI	Angioedema	Allele A—increased risk of angioedema in males	.03	Woodard-Grice et al. (2010)
*PRKCQ*	rs500766	g.6508628C>T	664 (175 cases, 489 controls)	African and European Americans	Ramipril	Angioedema	Allele T—reduced risk of angioedema	0.03	Pare et al. (2013)
*ETV6*	rs2724635	g.11747039G>A					Allele G—increased risk of angioedema	2.11 × 10^−4^	
*MME*	rs989692	g.155083576T>C					CT + TT genotypes are associated with increased risk of angioedema compared to CC	0.009	
*KCNMA1*	rs2253201	g.77596639G>A	5,063 (173 cases, 4,890 controls)	Northern European	Any ACEI or ARB	Angioedema	Minor alleles in *KCNMA1* variants—angioedema incidence	<5 × 10^−8^	Rasmussen et al. (2020)
	rs2253202	g.77596635G>A							
	rs2673471	g.77597565A>G							
	rs2619635	g.77598844G>A							
	rs2670121	g.77599131A>G							
	rs2673455	g.77599353C>G							
*F5*	rs6025	g.169549811C>T	1,066 (408 cases, 658 controls	Mixed	Enalapril, Ramipril, Or Losartan	Angioedema	Carrying any of these variants increases the risk of angioedema (OR = 2.64, CI 1.74–4.01)	1.53 × 10^−7^	Maroteau et al. (2020)
	rs200157005	g.169530994G>A						0.3727	
	rs149389480	g.169542338G>A						0.3727	
	rs143509841	g.169555304T>G						0.3727	
	rs140530655	g.169544331C>T						8.80 × 10^−8^	

^a^ The reference and alternative alleles in (Ref>Alt) are according to the variant annotation in the National Center for Biotechnology Information (NCBI), reference human genome (GRCh38.p14) (https://www.ncbi.nlm.nih.gov/).

ACEIs, angiotensin-converting enzyme inhibitors; ARBs, angiotensin receptor blockers; Ref, reference allele; Alte, alternative allele.

## 4 Thiazide and thiazide-like diuretics

**FIGURE 3:**
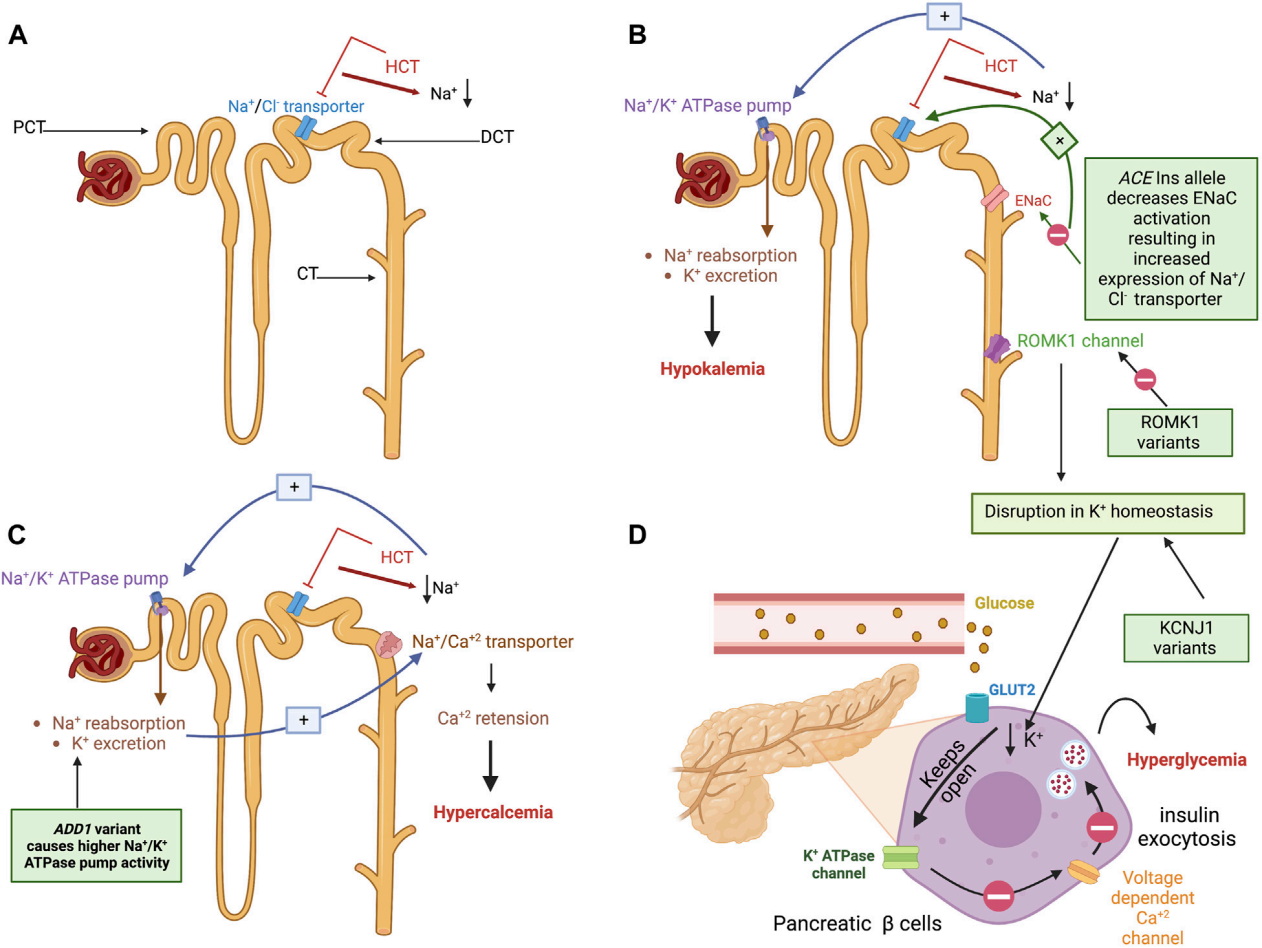
Hydrochlorothiazide mechanism of action, the pathogenesis of their metabolic side effects, and their proposed pharmacogenetic biomarkers. **(A)** An illustration of the nephrons proximal convoluting tubule (PCT), distal convoluting tubule (DCT), and collecting tubule (CT). HCT exerts its diuretic effect by inhibiting the sodium-chloride (Na^+^/Cl^−^) transporter in the DCT, thereby increasing sodium and chloride excretion. **(B)** Right: Mechanism of HCT-induced hypokalemia: To compensate for HCT-induced Na loss in the DCT, Na + concentration increases upstream in the CT. As a result, the renal aldosterone-sensitive sodium-potassium (Na^+^/K^+^) ATPase pump on the PCT is activated, promoting Na^+^ reabsorption and K^+^ excretion into CT and urine causing hypokalemia. Left: Pharmacogenetic variants associated with hypokalemia are illustrated in green boxes. The first is an *ACE* insertion hypothesized to exert its effect by affecting the epithelial Na^+^ channel (ENaC) responsible for Na^+^ and K^+^ transport. The reduced ENaC activation induced by an *ACE* insertion allele results in Na^+^ retention, increasing the Na^+^/Cl^−^ transporter. The latter will be inhibited by HCT, resulting in a profound effect of diuresis and higher HCT-induced potassium excretion. The other suggested variant affects the renal outer medullary potassium channel (ROMK1) in the CT, which is responsible for potassium excretion. Variants on *KCNJ1* influence the ROMK1 function and disrupt potassium homeostasis. **(C)** Mechanism of HCT-induced hypercalcemia: Due to the Na^+^ reabsorption resulting from the Na^+^/k^+^ transporter activation (described earlier), the Na^+^/Ca2^+^ transporter linked to the Na^+^/Cl^−^ transporter causes passive calcium influx into the lumen in distal tubule resulting in calcium retention and hypercalcemia. The green box shows the proposed mechanism of the *ADD1* variant’s contribution to exacerbating this adverse event by causing higher Na/K ATPase pump activity. **(D)** Mechanism of HCT-induced hyperglycemia: The disruption of potassium homeostasis decreases potassium in the interstitial fluid of the pancreatic β cells causing the K^+^ channels to remain open for an extended period resulting in cell hyperpolarization. The hyperpolarized cell will not be able to open voltage-gated calcium channels; thus, the intracellular calcium concentration will not increase, which in turn prevents the exocytosis of insulin granules resulting in decreased insulin secretion, hence hyperglycemia. *KCNJ1* variants cause potassium homeostasis disruption, which affects insulin secretion from pancreatic β cells or glucose uptake by skeletal muscles. (Figure created with BioRender.com).

Thiazide diuretics, including hydrochlorothiazide (HCT) and thiazide-like diuretics such as chlorthalidone, are medications mainly used for treating HTN. Moreover, they are used in HF, diabetes insipidus, and CKD. They exert their diuretic effect by inhibiting the sodium-chloride (Na^+^/Cl^−^) transporter in the distal convoluting tubule (DCT), thereby increasing sodium and chloride excretion (Figure 3A). These medications cause metabolic side effects such as hypokalemia, hyperglycemia, hypercalcemia, hyperlipidemia, and hyperuricemia (Akbari and Khorasani-Zadeh, 2022). Moreover, compared to other AHs, continuous usage of thiazides may result in an excess of 3%–4% of new-onset diabetes (NOD) (Minutolo et al., 2022).

To compensate for HCT-induced Na loss in the DCT, Na^+^ concentration increases upstream in the collecting tubule (CT). As a result, Na^+^ reabsorption and K^+^ excretion into CT and urine are promoted through the aldosterone-sensitive sodium-potassium (Na^+^/K^+^) ATPase pump on the proximal convoluting tubule (PCT), resulting in potassium loss (Ellison and Loffing, 2009; Akbari and Khorasani-Zadeh, 2022) (Figure 3B). Vormfelde and coworkers opted to test genetic variants and their effect on HCT blood pressure lowering effects in healthy individuals. The tested variants included common variants in *ACE*, alpha-adducin (*ADD1*), and G protein β subunit 3 (*GNB3*) genes. Although the results failed to prove an association with an AH response, it concluded that potassium excretion was significantly associated with the *ACE* Ins/Del polymorphism in a gene-dose-dependent manner. The Ins/Ins genotype carriers have higher K^+^ excretion than the Del/Del carriers. The authors suggested that the Ins/Ins carriers have less ACE activity, thus less angiotensin-II and aldosterone production (Vormfelde et al., 2006). Aldosterone activates epithelial Na^+^ channel (ENaC) in aldosterone-sensitive distal nephron (ASDN), causing Na^+^ retention and K^+^ excretion (Summa et al., 2001). Accordingly, The Ins/Ins genotype carriers will have less ENaC activation for Na^+^ retention, resulting in increased expression of Na^+^/Cl^−^ transporter, which further will be blocked by HCT, resulting in a profound effect of diuresis and higher thiazide-induced potassium excretion (Vormfelde et al., 2006).

Indeed, although hypokalemia abolishes the CV protective benefits of reducing blood pressure (Franse et al., 2000), very few studies investigated pharmacogenomic biomarkers for HCT-induced hypokalemia. A sub-analysis conducted on participants in the Pharmacogenomic Evaluation of Antihypertensive Responses (PEAR) study receiving HCT assessed the association between a variant in Potassium Inwardly Rectifying Channel Subfamily J Member 1 (*KCNJ1*), namely, rs59172778 (g.128839231A>G), and potassium levels. A significant association was observed between the “G” allele and increasing potassium levels in non-black patients. *KCNJ1* encodes the renal outer medullary potassium channel, a CT channel responsible for potassium excretion. The variant was hypothesized to disrupt the potassium homeostasis (Karnes et al., 2013). Figure 3B illustrates the mechanism of HTC-induced hypokalemia and its pharmacogenetic variants.

Moreover, Del-Aguila et al. (2015) conducted a GWAS of hypertensive patients treated with HCT with potassium level measurements to investigate genetic biomarkers of this side effect. The meta-analysis identified four variants with genome-wide significance: rs10845697, close to heme binding protein one gene (*HEBP1*), rs1007869 (g.87583313C>T) and rs12596186 (g.87582961G>A), close to junctophilin three gene (*JPH3*), and rs11135740 (g.23510244G>A), close to mitoferrin gene (*MFRN1*). The authors hypothesized that HCT affects blood K^+^ levels partially by influencing heme levels, which subsequently affect the K^+^ channel voltage. Nevertheless, the identified associations were not replicated in independent cohorts (Del-Aguila et al., 2015).

Interestingly, a recent approach to identifying HTN patients at high risk for developing hypokalemia invested in machine learning advances. This approach used data from over 25,000 HTN patients to create a predictive model pinpointing key hypokalemia-associated features. The authors proposed that their model streamlines the identification of at-risk patients in advance (Lin et al., 2023). However, incorporating genetic predictors into such models can enhance their predictive capacity. Such an approach has been preliminarily tested in the oncology (Larkin et al., 2023), and its extension to common diseases and widely used medications holds promise. Nonetheless, extensive research is required to select the most appropriate genetic biomarkers and evaluate machine learning-based models for clinical application.

Hypercalcemia is another diuretic-associated adverse event. Due to Na^+^/k^+^ transporter activation and Na^+^ reabsorption, the Na^+^/Ca^2+^ transporter causes passive calcium influx into the lumen and sodium excretion in DCT, resulting in calcium retention and hypercalcemia (Nijenhuis et al., 2005; Vormfelde et al., 2006). Alpha-adducin protein encoded by *ADD1* regulates Na+/k + pump signal transduction. This protein is involved in the spectrin-actin network, which contributes to cellular signal transduction and supports the plasma membrane (Kiang and Leung, 2018). Vormfelde et al. (2006) demonstrated an association between HCT-induced hypercalcemia and the *ADD1* rs4961 (g.2904980G>T) polymorphism. Individuals carrying alternative alleles exhibited higher calcium retention compared to carriers of the reference allele (Vormfelde et al., 2006). This association causes hypercalcemia by increasing the Na^+^/k^+^ pump activity, causing more Na^+^ reabsorption (Kiang and Leung, 2018). The proposed mechanism of HCT-induced hypercalcemia and the suggested role of ADD1 are depicted in Figure 3C. Indeed, no further studies investigated biomarkers of HCT-induced hypercalcemia. A review of the trends in hypercalcemia prevalence among HCT users over 2 decades indicated the high prevalence of this adverse event, despite its mild consequences (Griebeler et al., 2016). This might explain the relatively low level of interest in investigating it further.

Another adverse event induced by thiazides and thiazide-like diuretics is hyperglycemia and possibly increased NOD risk. This metabolic side effect is associated with the medications’ ability to induce hypokalemia. The decreased potassium in the interstitial fluid of the pancreatic β cells causes the K^+^ channels to remain open for an extended period, resulting in cell hyperpolarization. The hyperpolarized cell cannot open voltage-gated calcium channels; thus, the intracellular calcium concentration will not increase, preventing insulin granules’ exocytosis, resulting in decreased insulin secretion, hence hyperglycemia (Sica, 2004; Singh et al., 2018; Akbari and Khorasani-Zadeh, 2022). Figure 3D illustrates this adverse event mechanism.

Depending on this proposed mechanism, Karnes et al. (2013) investigated the effect of *KCNJ1* variants for associations with fasting glucose levels and NOD. The authors reported that rs17137967 (g.128847623T>C) was significantly associated with increased blood fasting glucose levels in black patients. In comparison, rs7933427 was nominally associated with this effect in non-black participants. On the other hand, allele “A” in rs675388 (g.128838114G>A) was associated with a 3.13-fold increase in NOD risk only for black participants, and allele “C” in rs12795437 (g.128860981G>C) and “A” in rs11600347 (g.128863419C>A) were significantly associated with 2- fold increase NOD risk for white participants. In contrast, allele “A” in rs658903 (g.128858172T>A) was associated with a greater than 60% reduction in NOD risk in Hispanics. Ethnic-stratified haplotype analysis revealed that a (GCA) haplotype at rs2238009 (g.128841142C>T), rs12795437 (g.128860981G>C), and rs11600347 (g.128863419C>A) SNPs, respectively, associated significantly with NOD risk in white patients. In Hispanics, a (CATCT) haplotype at rs675388 (g.128838114G>A), rs1148058 (g.128842886G>A), rs658903 (g.128858172T>A), rs12795437 (g.128860981G>C), and rs3016774 (g.128865771T>C), was associated with a 2-fold increased risk of NOD in contrast to (TGAGC) haplotype associated with a 57% decrease in NOD risk. Finally, in black patients, (GC) at rs675388 (g.128838114G>A)and rs1148059 (g.128843949C>G) was associated with a 72% decrease in NOD risk (Karnes et al., 2013). Despite the significant associations detected in this work, these were still significant after adjustment for serum potassium levels, indicating that the genetic variants are independent effectors. The authors proposed that although potassium is involved in hyperglycemia induction, the mechanism of this effect is more complex than a simple inverse correlation between glucose and K^+^ levels (Karnes et al., 2013).

One of the hypothesis-free thiazide pharmacogenomic studies investigated the genetic variants’ associations with thiazide-induced hyperglycemia through a GWAS. In this study, patients were receiving either HCT or chlorthalidone. A statistically significant association between the post-treatment increased blood glucose levels and two variants on two genes was detected among black patients. Firstly, rs9943291 (g.119749667T>G) on the 3-hydroxy-3- methylglutaryl-CoA synthase 2 (*HMGCS2*) gene, which encodes the HMGCS2 enzyme involved in ketogenesis and glucose regulation. This variant, the only one reaching genome-wide significance in the replication cohort, affects the expression (i.e., cis-eQTL effect) of the phosphoglycerate dehydrogenase (*PHGDH*) gene locally. *PHGDH* encodes the PHGDH enzyme involved in serine amino acid synthesis. Further studies are needed to determine how these variants interact and induce hyperglycemia (Singh et al., 2018). Secondly, rs61827877 (g.173823772C>G) in the intergenic region near *C1orf98*, which increases the expression (i.e., positive eQTL signal) of the zinc finger protein 281 (*ZNF281*) involved in the blood glucose regulation (Singh et al., 2018). Additionally, variants located on different genes were associated with increased blood glucose among black but not white patients post-chlorthalidone treatment only. These include; rs9927344 (g.12294252C>T) in sorting nexin 29 (*SNX29*) gene and rs201505549 (g.19743127_19743131del) in the Solute Carrier Family 4 Member 2 (*SLC24A2*) (Singh et al., 2018). The latter encodes the SLC24A2 transporter, which is a sodium-calcium-potassium exchanger. One recent study showed that this transporter is expressed in pancreatic β cells and may be involved in regulating the blood glucose (Bian et al., 2022).

Additionally, HCT is recognized for causing hyperuricemia and increased gout risk. HCT enters the PCT through the organic anion transporters OAT1 and OAT3 located on the basolateral membrane of proximal cells. These transporters are also responsible for uric acid (UA) excretion. HCT competes with UA for transporter, hindering UA binding and resulting in its accumulation and hyperuricemia (Kim and Jun, 2022). To our knowledge, only one GWAS was conducted to investigate the genetic contribution to HCT-induced hyperuricemia. Vandell and coworkers found variants on five genes (*LUC7L2*, *COX18/ANKRD17*, *FTO*, *PADI4,* and *PARD3B*) in African American and on (*GRIN3A*) in Caucasians, associated with increased UA levels. Following a meta-analysis, only *LUC7L2:*rs6947309 (g.139351084C>T) association with UA levels reached genome-wide significance in African Americans. Variants in the same gene and *PARD3B* were significantly associated with new-onset hyperuricemia in Black Americans. A risk score of variants in the five genes explained approximately 11% of UA variability following HCT treatment in African Americans (Vandell et al., 2014).

Finally, thiazide-induced dyslipidemia may occur in patients receiving high doses. Two mechanisms have been suggested; the thiazide-induced decrease in blood volume activates RAAS, which stimulates the release of epinephrine and norepinephrine, causing lipolysis and leading to hyperlipidemia (Sica, 2004; Del-Aguila et al., 2014). This effect is more profound in males, diabetic patients, and high diuretic doses (Sica, 2004). The second suggested mechanism involves decreased lipase enzyme activity due to impaired insulin production and secretion (Del-Aguila et al., 2014). Del-Aguila et al. (2014) conducted a GWAS that detected no significant associations with diuretic-induced hyperglycemia. In contrast, two SNPs, rs12279250 (g.21543533T>C) and rs4319515 (g.21556821T>C) located on the Neural EGFL Like 1 (*NELL1*) gene, were significantly associated with Thiazide-induced hypertriglyceridemia in African Americans. NELL1 protein suppresses adipogenic differentiation in unipotent preadipocytes and multipotent adipose-derived stromal cells, leading to lipogenesis repression (James et al., 2011; Del-Aguila et al., 2014). Accordingly, it is suggested that the differentiation of adipocytes through NELL1 may be modulated with HCT leading to triglyceride accumulation (Del-Aguila et al., 2014). More recently, a meta-analysis of various cohorts treated with either thiazides or loop diuretics was conducted to investigate any genetic variants’ effects on diuretic-induced dyslipidemia. The analysis, which included 6,787 patients on thiazides, yielded no significant genome-wide associations. The association of variants in the MACRO domain containing 2 gene (*MACROD2*) were the highest hit with HDL levels (de Las Fuentes et al., 2020).

Extensive and well-controlled studies are imperative due to the frequent occurrence of thiazide-induced metabolic adverse effects and their substantial impact on medication adherence. With the identified negative impact of hypokalemia on AHs’ CV protective effect (Franse et al., 2000), future research endeavors should emphasize understanding the mechanisms that underlie this effect. Additionally, deciphering the K^+^ imbalance mechanisms can contribute to understanding hyperglycemia and NOD. With advancements in whole exome/genome sequencing techniques, reliance solely on targeted genotyping might limit the potential findings. Moreover, merging the outcomes from such studies, which, as far as we know, have not been conducted extensively for thiazide-induced adverse events, with machine learning-generated models (Lin et al., 2023), holds the promise of establishing a safer utilization protocol for this widely used class of drugs.

## 5 Calcium channel blockers

CCBs are typically categorized into two groups: dihydropyridines (DHPs), exemplified by amlodipine, and non-dihydropyridines (non-DHPs), represented by verapamil and diltiazem. DHPs are mainly used as AHs, whereas non-DHPs find application in treating angina pectoris and dysrhythmias. CCBs act by binding to L-type voltage-gated calcium channels in vascular smooth muscle cells and cardiomyocytes. By doing so, they inhibit the inward flux of Ca^+2^ into the cells, thereby eliciting relaxation and vasodilation with DHPs, and slowing cardiac conduction and contraction with non-DHPs (Becker et al., 2009; McKeever and Hamilton, 2022). DHPs are generally well-tolerated, but they may induce side effects like flushing, excessive hypotension, peripheral edema, and reflex tachycardia (Taddei and Bruno, 2019).

Indeed, scarce data exist about the prevalence of CCBs’ adverse events and any genetic contribution to these events. Hyperglycemia and NOD have been frequently reported with the CCB amlodipine. The Genetics of Hypertension Associated Treatment (GenHAT) was designed to examine genetic components of HTN treatment response. Irvin et al. (2010) opted to look for associations of variants in the GenHAT-tested genetic variants with fasting glucose in patients receiving AHs from different classes, including amlodipine, lisinopril, or chlorthalidone. Tested variants were selected in the pathways proposed to interact with glucose metabolism, like RAAS and NO synthesis pathways. The authors reported two *ACE* variants, the aforementioned Ins/Del variants and the promoter variant rs4291 (g.63476833T>A), that showed significant associations with lower fasting glucose during AH treatment. The latter was the only one still significant after adjustment for multiple testing. There has been inconsistent evidence linking this allele to ACE concentration. One research reported that the minor allele is linked to higher plasma levels of ACE, although two other studies claimed the reverse. Nevertheless, in their investigation, Irvin et al. (2010) reported that the *ACE* variant has a small impact on fasting glucose during AH therapy and suggested further verification studies.

A recent meta-analysis discovered an association between the use of CCBs and glaucoma. This analysis involved over 140,000 European participants and aimed to identify commonly used medications linked to glaucoma and intraocular pressure. Due to the observational nature of this study, further research is necessary to ascertain a causal relationship between CCBs and glaucoma (Vergroesen et al., 2023). If a causative link is established for this association, the subsequent step would involve identifying any genetic variants that elevate the risk of its occurrence.

## 6 Beta-blockers

BBs act by blocking beta 1-adrenergic receptors (β1-ARs) in the heart, preventing endogenous catecholamines (epinephrine and norepinephrine) from binding and exerting their effects in decreasing heart rate and myocardial contraction. In addition, they reduce blood pressure by decreasing cardiac output and renin release. BBs are classified into two subgroups based on their selectivity: non-selective BBs and cardio-selective BBs. Non-selective BBs such as propranolol can bind to β1-ARs and β2-ARs, while Cardio-selective BBs such as atenolol specifically bind to β1-ARs (Farzam and Jan, 2022).

BBs are associated with higher rates of diabetes, dyslipidemia such as high triglycerides (TG), and bradycardia. Older BBs, such as propranolol and atenolol, lower blood pressure by reducing cardiac output, while peripheral vascular resistance is either maintained or elevated. Patients taking these BBs are more prone to dyslipidemia and suboptimal glycemic control. Contrarily, vasodilating BBs, such as labetalol and carvedilol, reduce blood pressure while decreasing peripheral vascular resistance, thereby boosting peripheral blood flow, which could lead to better tolerability and metabolic profiles. Some different mechanisms have explained the detrimental BBs’ effects on lipid and glucose metabolism. Firstly, the unopposed α1-adrenergic receptor activation can cause vasoconstriction, diminishing skeletal blood flow and lessening insulin-induced peripheral glucose absorption. Secondly, BBs may prevent pancreatic beta (β)-cells from secreting insulin. Moreover, weight gain is directly related to decreased insulin sensitivity and has been observed in people using conventional BBs (Deedwania, 2011).

Pharmacogenomic studies investigating any genetic component of BBs’ adverse events followed the targeted gene approach and GWAS design. One targeted gene approach study examined the variants identified earlier to be associated with fasting glucose and assessed their relationship with glucose levels in patients taking HCT or atenolol. The results showed an association between rs340874 (g.213985913T>C) in the 5′region of Prospero Homeobox 1 (*PROX1*) gene and atenolol-induce glucose elevation after 9 weeks of using the medication (Gong et al., 2014). *PROX1* encodes a transcription factor essential for tissue development, including endothelial lymphatic vessels, the liver, and the pancreas. In addition, PROX1 is expressed in endocrine islets and appears to be associated with pancreatic progenitor cell differentiation and proliferation. Indeed, the inhibition of PROX1 expression was linked to a roughly 2-fold decrease in glucose-stimulated insulin secretion, giving a biological relevance to the detected association (Gong et al., 2014).

Another study examined genes active in the melatonin pathway, which regulates insulin secretion and glucose homeostasis (Chang et al., 2016). Melatonin acts through melatonin receptors 1 and 2 (MT1 and MT2). The transcription of these receptors is inversely proportional to melatonin plasma levels, which are controlled by the circadian rhythm. Both receptors are expressed in pancreatic β cells and play a role in regulating insulin secretion (Mok et al., 2019). Given their significance in glucose regulation, Chang et al. (2016) investigated genes active in the melatonin pathway and the vulnerability to glucose dysregulation post-atenolol treatment. The authors reported that the variant allele at rs11649514 (g.24175240G>T) on the Protein Kinase C Beta (*PRKCB*) was significantly associated with increased glucose levels. *PRKCB* encodes protein kinase C (PKC), which is involved in the Gq/11 melatonin signal transduction pathway to increase insulin secretion. In addition, the authors reported that atenolol monotherapy caused an overall increase in fasting glucose and decreased urinary 6-sulfatoxymelatonin (aMT6s) levels, a melatonin metabolite, without proof of a connection between the two alterations (Chang et al., 2016).

Furthermore, an integrative approach of metabolomic and genomic variants identified the baseline β-alanine levels associated with glucose levels. Further analysis of genetic data pointed out an association signal at rs2669429 (g.104451462A>G) located on the dihydropyrimidinase (*DPYS*) gene. The minor allele carriers had significantly higher glucose levels post-atenolol treatment. *DPYS* encodes for the DHP enzyme, which is involved in β-alanine formation. The latter is a non-essential amino acid involved in the formation of carnosine. The findings suggest that carnosine derived from β-alanine plays a part in the dysregulation of glucose metabolism following treatment with a BB. The authors suggested that *DPYS*:rs2669429 and β-alanine levels may be useful biomarkers for identifying individuals more likely to develop diabetes and hyperglycemia during atenolol treatment (de Oliveira et al., 2016).

Indeed, essential amino acids (AAs), including aromatic amino acids (AAA), and branched-chain amino acids (BCAA), are significant and independent risk biomarkers for diabetes and insulin resistance in young adults. The low levels of these amino acids can be caused by polymorphisms in genes encoding phenylalanine hydroxylase (*PAH*) and branched chain amino-acid transaminase 1 (*BCAT1*), responsible for metabolizing AAA and BCAA, respectively (Cooper-DeHoff et al., 2014). It was found that altered BCAA and AAA metabolism is linked to impaired insulin sensitivity (Würtz et al., 2013; Fontana et al., 2016). Cooper-DeHoff et al. (2014) hypothesized that baseline levels of BCAA and AAA, or genetic variants affecting their catabolic pathways, can increase fasting glucose after short-term atenolol exposure. This study identified a *PAH* variant, rs2245360 (g.102840766G>A), with a statistically significant association with increased fasting glucose levels. In addition, there was a significant association between the levels of these AAs and the odds of developing post-atenolol-increased fasting glucose. The authors suggested that if a patient has metabolic risk factors, atenolol could be an environmental trigger (Cooper-DeHoff et al., 2014). However, this study and the previous ones were limited by their small sample size and included only Americans of European ancestry.

In contrast to the targeted gene approach, Chang and coworkers applied a GWAS to explore genetic variants associated with atenolol-induced hyperglycemia. This study recruited patients treated with either atenolol or verapamil (N = 232). Allele “G” of rs11124945 (g.43650017A>G) was significantly associated with a lower risk for new-onset diabetes (NOS). In contrast, in homozygous carriers, the “A” allele was associated with an increased risk for NOS when treated with atenolol. These findings imply that for allele “G” carriers, BB-based regimens would be preferable to CCB-based regimens and *vice versa*. This variant is in the Pleckstrin Homology Domain-Containing Family H Member 2 (*PLEKHH2*), involved in the attachment of kidney podocytes to the basement membrane and actin stability. SNPs within *PLEKHH2* were previously linked to diabetic nephropathy. Moreover, *PLEKHH2:* rs11124945 is almost 50 kb upstream of the thyroid adenoma-associated (*THADA*) gene, which has been linked to type 2 diabetes for many years. *THADA* is involved in apoptosis and death receptor signaling, and its variants are thought to impair β-cell function by decreasing the cell’s mass. Herein, the 2p21 locus contains both *PLEKHH2* and *THADA*, associated with DM, and has functional evidence for controlling gene expression (Chang et al., 2018).

Furthermore, BBs have adverse effects on the patient’s lipid profile, causing hypercholesterolemia and hypertriglyceridemia, an effect that is more prevalent with Non-selective BB. However, pancreatitis has been reported in patients treated with either group, as both potentially exacerbate already present hypertriglyceridemia (Iaccarino et al., 2005; Tziomalos et al., 2011). The mechanism underlying these adverse events may be intuitive, given the presence of β-adrenergic receptors (AR) in the liver and their role in regulating glucose and lipid homeostasis. One study assessed two common variants of the Adrenoceptor Beta 2 (*ADRB2*) gene, known for their effect on BB response. It showed that the variant allele *ADRB2*:rs1042714 (g.148826910G>C) was associated with increased dyslipidemia incidence. Patients carrying this allele showed increased TG serum levels post atenolol and metoprolol treatment. *ADRB2* encodes β2-Ars, which play a role in lipolysis regulation in skeletal muscles. Since this variant is a gain-of-function variant that increases β2-AR signaling, it is speculated that the increased lipolysis causes subsequent hypertriglyceridemia. It is also possible that the β1-AR blockage may result in the preferential activation of β2-AR, explaining why BB treatment is associated with an increased incidence of dyslipidemia in patients with this variant (Iaccarino et al., 2005; Isaza et al., 2007).

Like other medications, some BBs are affected by Cytochrome P450 (CYP) enzymes. CYP2D6 accounts for 70%–80% of metoprolol metabolism but contributes less to metabolizing carvedilol, nebivolol, and propranolol. Other BBs, such as atenolol, are unaffected by CYP2D6 as they are mainly eliminated unchanged through kidneys. The phenotype of CYP2D6 is determined by the activity score (AS), which assigns an “activity value” to each allele. Given that AS >2.25 is an ultra-metabolizer (UM), AS 1.25 ≤ x ≤ 2.25 is considered a normal metabolizer (NM), and AS = 0 is regarded as a poor metabolizer (PM) (Bijl et al., 2009; Caudle et al., 2020). Multiple studies investigated the effect of CYP2D6-impaired alleles on heart rate for BB users. One of the studies conducted for this purpose investigated the association among 1,533 patients receiving different BB medications (metoprolol, atenolol, and bisoprolol). A statistically significant association was found between CYP2D6*4 and heart rate in patients receiving metoprolol. Patients on metoprolol carrying this allele had a considerably higher risk (odds ratio 3.86) of bradycardia than NMs (Bijl et al., 2009). The Dutch Pharmacogenomics Work Group (DPWG) released metoprolol-*CYP2D6* pharmacogenomic-guided guidelines in 2018 and updated them in 2022 (Dean, 2012). Nevertheless, these guidelines do not recommend testing for *CYP2D6* alleles before metoprolol use or applying any changes in dosing unless the occurrence of symptomatic bradycardia.

Indeed, the effect of *CYP2D6* alleles and enzyme activity with metoprolol-induced bradycardia has inconsistent evidence, resulting from small study sizes or poor design. Nevertheless, this situation is expected to improve with the advances in preemptive pharmacogenomic testing. For instance, at Sanford Health, Collett et al. (2023) retrospectively analyzed data from patients with preemptive pharmacogenomic profiles, including *CYP2D6*. The authors identified 114 poor CYP2D6 PMs prescribed metoprolol, matched with 114 NMs, and reported a significant difference between the two groups in the incidence of bradycardia. Despite the limitations of this study, related to its retrospective nature, not controlling for metoprolol dose or formulation, and being a result of one center with a single population inclusion, it adds to the body of literature on this relationship, (Collett et al., 2023), and illustrates the potential benefits of preemptive pharmacogenomic testing in actual life practice.

Finally, using data from different randomized clinical trials, a GWAS meta-analysis investigated the association between individual AH classes and adverse cardiovascular outcomes. All the included studies were on Caucasians treated with other AHs. The most significant association was between the “T” allele of rs139945292 (g.131320708C>T) and an increased adverse cardiovascular risk in patients treated with BBs and a decreased risk in patients treated with other AHs. This SNP is located 50 kb upstream of the Neurotrimin (*NTM*) gene, which encodes one of the cell adhesion molecules and is mainly expressed in the atrial appendage of the heart. The authors suggested that the alternative allele increases the expression of NTM, which may attenuate BP response to BBs, leading to increased adverse cardiovascular risks (McDonough et al., 2021).

In summary, despite the multiple attempts to find a biomarker for BB adverse events, none of the research identified a reproducible association, except for the growing evidence of *CYP2D6* and metoprolol-associated bradycardia. The availability of large cohorts with endless possibilities for analyzing data at different levels should enable a better understanding of BB adverse events’ molecular mechanisms. Such understanding can contribute to better use of these drugs in the future. Specifically, BB-induced hyperglycemia should be prioritized, given that current evidence provides a decent understanding of its mechanisms that need more rigorous confirmation efforts.

Table 2 summarizes the prominent research efforts that yielded significant associations between pharmacogenetic variants (Genes and variants are listed in Supplementary Tables S1, S2) and adverse events induced by thiazide and thiazide-like diuretics, CCBs, and BBs.

**TABLE 2:** Studies investigating pharmacogenomic biomarkers associated with thiazide, CCBs, and BBs-induced adverse events.

Gene	rs ID	^a^Ref > Alt	Sample size	Ethnicity	Medication	Studied outcome	Genotype/Allele-associated effect	*p*-value	Reference
*KCNJ1*	rs17137967	g.13031952G>A/T	768 from “PEAR” cohort And 410 from “INVEST” cohort	Mixed	Hydrochlorothiazide	Dysglycemia	CC + CT—increased fasting glucose in blacks	.009	Karnes et al. (2013)
	rs12795437	g.128860981G>C					Allele C—greater than 2-fold increase in NOD in Whites	.04	
	rs11600347	g.128863419C>A					Allele A—greater than 2-fold increase in NOD in Whites	.04	
	rs675388	g.128838114G>A					Allele A—greater than 3.13-fold increase in NOD in Blacks	.03	
	rs658903	g.128858172T>A					Allele A—>60% reduced risk of NOD in Hispanics	.04	
*HMGCS2*	rs9943291	g.119749667T>G	310 discovery and 362 replication cohort	Mixed	Chlorthalidone or Hydrochlorothiazide	Dysglycemia	Allele G—increased glucose levels	4.17 × 10^−8^	Singh et al. (2018)
69 kb 3′ of *LINC00862*	rs61824877	g.173823772C>G					Allele A—increased fasting glucose levels	4.82 × 10^−9^	
*SLC24A2*	rs201505549	g.19743127_19743131del					Deletion—increased fasting glucose levels	2.11 × 10^−9^	
*SNX29*	rs9927344	g.12294252C>T					Allele T—increased fasting glucose levels	3.86 × 10^−8^	
*LUC7L2*	*rs6947309*	g.139351084C>T	768 from “PEAR” cohort	Mixed	Hydrochlorothiazide	Hyperuricemia	Allele T-increased uric acid elevation in African Americans	7.18 × 10^−8^	Vandell et al. (2014)
*NELL1*	rs12279250	g.21543533T>C	768 from “PEAR” cohort	Mixed	Hydrochlorothiazide	Dyslipidemia	Allele C—increased plasma triglycerides levels	6.6 × 10^−9^	Del-Aguila et al. (2014)
	rs4319515	g.21556821T>C					Allele C—increased plasma triglycerides levels	4.05 × 10^−9^	
*ACE*	rs4291	g.63476833T>A	9,309 from the “GenHAT” cohort	Mixed	Lisinopril, Chlorthalidone, or Amlodipine	Dysglycemia	Allele A—decreased fasting glucose	0.001	Irvin et al. (2010)
*PROX1*	rs340874	g.213985913T>C	456 from “PEAR” cohort	Caucasians	Atenolol or Hydrochlorothiazide	Dysglycemia	Allele C—increased fasting glucose levels after 9 weeks of atenolol therapy	0.0013	Gong et al. (2014)
*PAH*	rs2245360	g.102840766G>A	234 from the “PEAR” cohort	Caucasians	Atenolol	Dysglycemia	AA genotype—impaired fasting glucose levels	0.0003	Cooper-DeHoff et al. (2014)
*PRKCB*	rs11649514	g.24175240G>T	232 from the “PEAR” cohort	Caucasians	Atenolol	Dysglycemia	GT + TT genotypes -increased fasting glucose levels	1.0 × 10^−4^	Chang et al. (2016)
*DPYS*	rs2669429	g.104451462A>G	234 from the “PEAR” cohort	Caucasians	Atenolol	Dysglycemia	Allele G—increased fasting glucose levels	0.0006	de Oliveira et al. (2016)
*PLEKHH2*	rs11124945	g.43650017A>G	1,140	Mixed	Atenolol or verapamil	Dysglycemia	Allele G—lower odds for NOD when exposed to the β-blocker than CCB	4.02 × 10^−5^	Chang et al. (2018)
			334 NOD cases and 806 controls						
*ADRB2*	rs1042714	g.148826910G>C	105	South American with Mestizo origins	Metoprolol	Dyslipidemia	CG genotype—increased triglycerides levels	0.025	Isaza et al. (2007)
*CYP2D6*	rs3892097	g.42128945C>A	1,533 from “Rotterdam cohort”	Europeans	Metoprolol	Bradycardia	CYP2D6^a^4–increased bradycardia risk	0.0014	Bijl et al. (2009)

^a^ The reference and alternative alleles in (Ref > Alt) are according to the variant annotation in the National Center for Biotechnology Information (NCBI), reference human genome (GRCh38.p14) (https://www.ncbi.nlm.nih.gov/). BBs, beta blockers; CCB, calcium channel blockers; DHP-CCBs, dihydropyridine calcium channel blockers; INVEST, international verapamil-trandolapril study; PEAR, pharmacogenomic evaluation of antihypertensive responses; NOD, new onset of diabetes; GenHAT, the genetics of hypertension associated treatment.

## 7 Conclusion and perspectives

The current review represents the first comprehensive focus on pharmacogenomic variants associated with adverse effects of AH medications rather than their response. We highlighted evidence supporting the association of *ACE* variants with adverse effects such as ACEIs-cough or AE, as well as hyperglycemia induced by various AH drugs. Genes actively involved in the BK pathway are also recommended to be prioritized for examining adverse effects of ACEIs and ARBs. Furthermore, the metabolic adverse events provoked by thiazide and thiazide-like diuretics, as well as BB-associated hyperglycemia, warrant further investigation, particularly focusing on genes involved in regulating potassium balance pathways.

Except for the DPWG metoprolol-induced bradycardia-related guidelines, no other guidelines have been emphasized by the Clinical Pharmacogenetics Implementation Consortium (CPIC), American Heart Association (AHA), American College of Cardiology (ACC) and European Society of Cardiology (ESC) regarding the integration of genotype-guided dosage for antihypertensive medications within the healthcare systems as with other cardiovascular medicines such as warfarin and clopidogrel which have successfully genotype-guided dosage implementation. Accordingly, currently the antihypertensive medicines chosen are based on clinical expertise and hypertension recommendations and guidelines (Eadon et al., 2018; Guidelines and Clinical Documents, 2022; Xiao et al., 2022; CPIC, 2023; European Society of Cardiology, 2023).

Unfortunately, numerous associations highlighted in this review have shown limited consistency and reproducibility. The challenges encountered in these studies mirror the common obstacles faced in pharmacogenomics research and implementation in general: small sample sizes, interethnic variations in the distributions of genetic polymorphisms leading to findings that are challenging to generalize and using targeted gene approaches rather than agonistic ones. Future research should invest in technological progress besides the unprecedented advances in data analysis. Exploring the potential of machine learning approaches, an aspect we briefly mentioned in this review, could be a valuable approach for providing more evidence to implement pharmacogenomics-based AHs therapies.

Considering the global utilization of AHs across diverse populations, engaging in international collaborative pharmacogenomic research could significantly expedite the identification of dependable biomarkers. This approach could pave the way for personalized AHs therapy, reducing adverse events and enhancing medication adherence on a broader scale.
