# Supplementary material for: Antihypertensives associated adverse events: a review of mechanisms and pharmacogenomic biomarkers available evidence in multi-ethnic populations
Source: Front Pharmacol. 2023 Dec 1;14:1286494. doi: 10.3389/fphar.2023.1286494 (PMC10722273; doi:10.3389/fphar.2023.1286494)
Supplement: Supplementary file 1 [file Table1.DOCX]

**Supplementary Material**

**Antihypertensives Associated Adverse Events: A Review of Mechanisms and Pharmacogenomic Biomarkers Available Evidence in Multi-Ethnic Populations**

Sahar M. Altoum^1^, Zeina N. Al-Mahayri^1^, Bassam R. Ali^1,2*^

**Table S1: The NCBI Reference Sequence accession and version numbers of genes mentioned in the text.**

| **Gene** | **RefSeq (NCBI)** |
| --- | --- |
| *BDKRB2* | NM_000623.4* |
| *ACE* | NG_011648.1 |
| *SLCO1B1* | NG_011745.1 |
| *ABO* | NG_006669.2 |
| *KCNIP4* | NG_052969.1 |
| *XPNPEP2* | NG_011479.2 |
| *PRKCQ* | NM_006257.5* |
| *ETV6* | NG_011443.1 |
| *MME* | NG_051105.1 |
| *KCNMA1* | NG_012270.1 |
| *F5* | NG_011806.1 |
| *F12* | NG_007568.1 |
| *ADD1* | NG_012037.1 |
| *GNB3* | NG_009100.2 |
| *KCNJ1* | NG_009379.1 |
| *HEBP1* | NM_015987.5* |
| *JPH3* | NG_009797.1 |
| *MFRN1 (SLC25A37)* | NM_016612.4* |
| *HMGCS2* | NG_013348.1 |
| *PHGDH* | NG_009188.1 |
| *C1orf98* | NR_040064.1** |
| *ZNF281* | NM_012482.5* |
| *SNX29* | NM_032167.5* |
| *SLC24A2* | NG_029734.2 |
| *LUC7L2* | NG_030012.1 |
| *COX18* | NM_001297732.2* |
| *ANKRD17* | NM_032217.5* |
| *FTO* | NG_012969.2 |
| *PADI4* | NG_023261.3 |
| *PARD3B* | NM_001302769.2* |
| *GRIN3A* | NM_133445.3* |
| *NELL1* | NG_047064.1 |
| *MACROD2* | NG_054905.1 |
| *PROX1* | NM_001270616.2* |
| *PRKCB* | NG_029003.2 |
| *DPYS* | NG_008840.2 |
| *PAH* | NM_000277.3* |
| *BCAT1* | NM_005504.7* |
| *PLEKHH2* | NM_172069.4* |
| *THADA* | NM_001032896.2* |
| *ADRB2* | NG_016421.2 |
| *CYP2D6* | NG_008376.4 |
| *NTM* | NM_016522.3* |

* For genes represented by individual transcript accessions rather than the gene locus, the accession number of transcript variant 1 is mentioned.

**Intergenic non-protein coding region

**Table S2: Genomic coordinates of variants mentioned in the text.**

| **Variant** | **Variant locus (GRCh38)** |
| --- | --- |
| rs1799722 | g.96204802C>T |
| rs1799752 | g.63488543_63488544insTTTTTTTTTTTGAGACGGAGTCTCGCTCTGTCGCCCATACAGTCACTTTT |
| rs4459610 | g.63507359A>T |
| rs4267385 | g.63506395C>T |
| rs4149056 | g.21178615T>C |
| rs495828 | g.133279294T>G |
| rs145489027 | g.21389571G>A |
| rs7661530 | g.21349637T>C |
| rs6838116 | g.21354260A>C |
| rs7675300 | g.21383914C>A |
| rs16870989 | g.21385141T>A |
| rs1495509 | g.21391993T>C |
| rs3788853 | g.129736814C>A |
| rs2050011 | g.129737601G>T |
| rs500766 | g.6508628C>T |
| rs2724635 | g.11747039G>A |
| rs989692 | g.155083576T>C |
| rs2253201 | g.77596639G>A |
| rs2253202 | g.77596635G>A |
| rs2673471 | g.77597565A>G |
| rs2619635 | g.77598844G>A |
| rs2670121 | g.77599131A>G |
| rs2673455 | g.77599353C>G |
| rs6025 | g.169549811C>T |
| rs200157005 | g.169530994G>A |
| rs149389480 | g.169542338G>A |
| rs143509841 | g.169555304T>G |
| rs140530655 | g.169544331C>T |
| rs59172778 | g.128839231A>G |
| rs10845697 | g.13031952G>A/T |
| rs1007869 | g.87583313C>T |
| rs12596186 | g.87582961G>A |
| rs11135740 | g.23510244G>A |
| rs4961 | g.2904980G>T |
| rs17137967 | g.128847623T>C |
| rs7933427 | g.128856082G>A/T |
| rs675388 | g.128838114G>A |
| rs12795437 | g.128860981G>C |
| rs11600347 | g.128863419C>A |
| rs658903 | g.128858172T>A |
| rs2238009 | g.128841142C>T |
| rs1148058 | g.128842886G>A |
| rs3016774 | g.128865771T>C |
| rs1148059 | g.128843949C>G |
| rs9943291 | g.119749667T>G |
| rs61827877 | g.173823772C>G |
| rs9927344 | g.12294252C>T |
| rs201505549 | g.19743127_19743131del |
| rs6947309 | g.139351084C>T |
| rs12279250 | g.21543533T>C |
| rs4319515 | g.21556821T>C |
| rs4291 | g.63476833T>A |
| rs340874 | g.213985913T>C |
| rs11649514 | g.24175240G>T |
| rs2669429 | g.104451462A>G |
| rs2245360 | g.102840766G>A |
| rs11124945 | g.43650017A>G |
| rs1042714 | g.148826910G>C |
| rs139945292 | g.131320708C>T |
